# Different Effect Sizes of Motor Skill Training Combined with Repetitive Transcranial versus Trans-Spinal Magnetic Stimulation in Healthy Subjects

**DOI:** 10.3390/brainsci14020165

**Published:** 2024-02-06

**Authors:** Farsin Hamzei, Alexander Ritter, Kristin Pohl, Peggy Stäps, Eric Wieduwild

**Affiliations:** 1 Section of Neurological Rehabilitation, Hans-Berger-Hospital of Neurology, Jena University Hospital, Am Klinikum 1, 07747 Jena, Germany; alexander.ritter@uni-jena.de (A.R.); kristin.pohl@moritz-klinik.de (K.P.); ericwieduwild@gmx.de (E.W.); 2 Department of Neurology, Moritz Klinik, Hermann-Sachse-Straße 46, 07639 Bad Klosterlausnitz, Germany; peggy.staeps@moritz-klinik.de

**Keywords:** non-invasive brain stimulation, motor skill training, repetitive transcranial magnetic stimulation, repetitive trans-spinal stimulation

## Abstract

Repetitive transcranial magnetic stimulation (rTMS) is used to enhance motor training (MT) performance. The use of rTMS is limited under certain conditions, such as after a stroke with severe damage to the corticospinal tract. This raises the question as to whether repetitive trans-spinal magnetic stimulation (rSMS) can also be used to improve MT. A direct comparison of the effect size between rTMS and rSMS on the same MT is still lacking. Before conducting the study in patients, we determined the effect sizes of different stimulation approaches combined with the same motor training in healthy subjects. Two experiments (E1 and E2) with 96 subjects investigated the effect size of combining magnetic stimulation with the same MT. In E1, high-frequency rTMS, rSMS, and spinal sham stimulation (sham-spinal) were applied once in combination with MT, while one group only received the same MT (without stimulation). In E2, rTMS, rSMS, and sham-spinal were applied in combination with MT over several days. In all subjects, motor tests and motor-evoked potentials were evaluated before and after the intervention period. rTMS had the greatest effect on MT, followed by rSMS and then sham-spinal. Daily stimulation resulted in additional training gains. This study suggests that rSMS increases excitability and also enhances MT performance. This current study provides a basis for further research to discover whether patients who cannot be treated effectively with rTMS would benefit from rSMS.

## 1. Introduction

The corticospinal tract (CST) originates from neurons in the premotor and primary motor cortex and terminates in the spinal cord, where it synapses with a neuron in the anterior horn of the spinal grey matter, allowing for muscle control from the opposite side of the brain [1]. Repetitive transcranial magnetic stimulation (rTMS) is a non-invasive approach that delivers a series of magnetic stimuli to specific areas of the brain to alter neuronal plasticity. By delivering a series of magnetic stimuli to targeted brain regions, inhibitory effects on motor cortical excitability are induced with a low-frequency (≤1 Hz) stimulation series, or cortical excitability is enhanced with a high-frequency (≥3 Hz) stimulation approach. Recent studies have shown that high-frequency rTMS over the primary motor cortex increases excitability and promotes motor learning [2,3]. Repetitive series of magnetic stimulation can also be applied peripherally to influence mechanisms of neuroplasticity. To improve a functional deficit, repetitive magnetic stimulation is also applied to the peripheral nerve [4], over muscle [2,3], or trans-spinal [5], defined as repetitive peripheral magnetic stimulation (rPMS). It has been shown that rPMS applied to the peripheral nerve [6], over muscle [7], and trans-spinal [8] increases cortical excitability.

As a combination of rTMS and motor training is more beneficial than motor training or rTMS alone [2], it is therefore of interest to see to what extent different stimulation options influence motor training. To date, a direct comparison between different stimulation options in combination with standardized motor training is lacking. Therefore, we were interested in analyzing the effect sizes of two stimulation sites of cortical (rTMS) and trans-spinal (repetitive trans-spinal magnetic stimulation: rSMS), in combination with motor training. We, therefore, carried out two experiments. 

In Experiment I (E1), the stimulation was combined once with the same motor training. In E1, four groups were formed. All groups practiced the same motor skill in a standardized way. One group additionally received rTMS (cortical impact), another group received rSMS (spinal interference), the third group received spinal sham stimulation (sham-spinal), and the last group received no stimulation and only practiced the motor skill. The aim of E1 was to find out whether rSMS stimulation is more beneficial than sham-spinal and motor training without stimulation. We hypothesized that the rSMS group would improve their motor skill performance more than both the sham-spinal group and the no-stimulation group. 

Since in neurorehabilitation, an intervention is applied over several days to weeks, in Experiment II (E2), we applied an intervention over several days. Three groups received either rTMS, rSMS, or spinal sham stimulation (sham-spinal) over four days in combination with standardized motor skill training. We hypothesized that motor skills would improve more with rTMS than with rSMS and that both would be superior to spinal sham stimulation (sham-spinal).

This study was carried out in healthy subjects because no study has yet compared both stimulation methods with the same motor training, so their effect sizes are still unknown. Therefore, before conducting the study in patients, we determined the effect sizes of different stimulation approaches combined with the same motor training in healthy subjects.

## 2. Materials and Methods

### 2.1. Trial Design and Participants

Experiment I (E1) and Experiment II (E2) are investigator-initiated, interventional, randomized, controlled efficacy studies. 

In Experiment I (E1), 60 healthy right-handed participants (mean age 23.6 ± 2.7 years; range between 20 and 30 years, 33 females) were included, and in Experiment II (E2), 36 healthy right-handed participants (mean age 23.5 ± 2.4 years; range between 20 and 30 years, 17 females) were included. Handedness was assessed using the ten-item Edinburgh Handedness Inventory [9]. Participants with a laterality quotient outside the range of 0.5–1.0 were excluded. Other exclusion criteria were any neurological or psychiatric disorder, arrhythmia, any central nervous system disorder (e.g., stroke, brain injury, head trauma, epilepsy), and metal in or on the body (e.g., pacemaker, cochlear implant, according to [10]). Subjects were also excluded if they were taking any medication.

The study was conducted in the laboratories of the Moritz Klinik. Subjects were recruited through advertisements in various information centers, for example, at the university. Interested subjects were contacted and informed about the study procedures. If they were still interested, they were asked about the inclusion and exclusion criteria, and their eligibility was checked.

All participants gave written informed consent before participating in the experiment. This study was conducted in accordance with the tenets of the Declaration of Helsinki and was approved by the local ethics committee (No. 5453-02/18).

### 2.2. Experimental Procedure Experiment I (E1)

**Figure 1:**
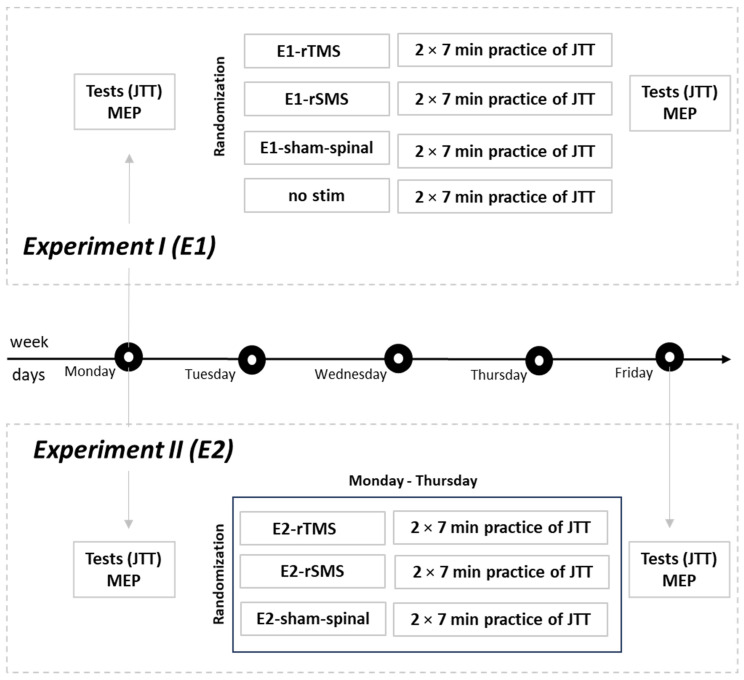
Experimental design of Experiment I (E1) and Experiment II (E2). In E1, stimulation and training took place on Mondays. In E2, the first stimulation and training session took place on Monday and was repeated daily from Tuesday to Thursday, with testing on Friday.

Subjects were first informed about the study, and their eligibility was checked. After approval, all subjects completed a motor test. The motor test used was the Jebsen–Taylor Hand Function Test (JTT, pre-test) [11]. Afterwards the motor-evoked potential (MEP) was then elicited in the first dorsal interosseous muscle (FDI) by transcranial magnetic stimulation (TMS). Subjects were then randomized into four groups: rTMS group (E1-rTMS); rSMS group (E1-rSMS); spinal sham stimulation group (E1-sham-spinal), and a group without stimulation (a no-stimulation group: no-stimulation). The intervention was administered immediately before motor skill training with JTT content. All subjects practiced the JTT twice for 7 minutes (min) with a 2 min break after 7 min of practice (net practice time, 14 min; total practice time, 16 min). At the end of the training period, the JTT was tested again (post-test). Following this, MEP was measured. Tests were performed with the right and left hand, and the training was performed with the left hand (Figure 1). Follow-up testing after three months was planned but could not be completed due to restrictions imposed by the Corona pandemic. These data have therefore been omitted.

### 2.3. Experimental Procedure Experiment II (E2)

Analogous to E1, subjects were first informed about the study, their eligibility was checked, and, after approval, they completed a motor test (JTT), and their MEPs were measured (pre-test). Subjects were then randomly assigned to three groups: rTMS group (E2-rTMS); rSMS group (E2-rSMS); and spinal sham stimulation group (E2-sham-spinal). Experiment II did not incorporate a group without stimulation, as mentioned in E1, due to the superior outcomes observed in all other groups compared to the no-stimulation group following the evaluation of the E1 results. The stimulation was performed immediately before the motor training of the JTT on Monday (after the motor test and after the MEP measurement), Tuesday, Wednesday, and Thursday. On Friday, there was no stimulation and no training, and the JTT was tested again followed by MEP measurement (post-test) (Figure 1). Similar to E1, all subjects practiced the JTT twice for 7 min with a 2 min break after 7 min of practice (net practice time, 14 min; total practice time, 16 min). The test was performed with both the right and left hand, and the practice was performed with the left hand. 

### 2.4. Motor Tests

The JTT has been used to measure unimanual hand function in various activities of daily living. The JTT consists of several subtests (writing a sentence, turning cards, picking up small objects, picking up kidney beans with a spoon, stacking checkers, placing empty cans on a board, placing full cans on a board) [11]. Writing a sentence was removed from the analysis of the JTT to avoid bias between native and non-native speakers of German. The JTT was performed with the non-dominant left hand followed by the dominant right hand. Time was recorded for each subtest and added up for each hand separately.

### 2.5. Motor Training

For motor training, all participants in E1 and E2 performed subsets of the JTT with their left, non-dominant hand only. The non-dominant left hand was chosen as the training site to avoid a ceiling effect of motor training. Each subtest of the JTT was practiced for one minute with no break between each subtest. After the first training session of 7 min, a break of 2 min was taken. Immediately afterwards, a second run was performed using the same procedure. For E1 and E2, the total training time was 16 min (two 7 min training sessions with a 2 min break in between).

### 2.6. MEP

Magnetic stimulation was delivered using a biphasic magstim^®^ rapid^2^ stimulator (The Magstim Company Ltd., Spring Gardens, Whitland, UK) discharging via a figure-of-eight coil (70 mm diameter) to determine the resting motor threshold (RMT) and to measure MEP (amplitude and latency). The EMG was recorded at the first dorsal interosseous muscle (FDI) of the left hand using Ag-AgCl electrodes (20 × 26 mm), which were connected with crocodile clips. During the measurement, participants were seated in a chair with their back and arms at rest. They were instructed to relax their left arm completely (from shoulder to hand). Participants wore a silicone swim cap on their head to reduce slippage of the coil when the optimal position was found. The coil was positioned to obtain the maximum compound MEP of the contralateral left FDI [12]. This position (called the hotspot) was defined approximately 5 cm lateral to the right vertex (Cz) according to the 10–20 system. The coil was oriented in a posterolateral–anteromedial direction at an angle of 45° to the midline [13]. The resting motor threshold (RMT) was defined as the lowest stimulation intensity that elicited at least five MEPs with an amplitude of at least 50 μV amplitude out of 10 consecutive stimulations [14]. The optimal stimulation site was defined as the hotspot where the largest MEP could be evoked consistently. The MEP latency of the target muscle was measured as the time in ms between stimulus onset and MEP onset. The amplitude was calculated as the peak-to-peak distance in mV. MEP amplitude and latency were recorded using neurowerk© (SIGMA Medizin-Technik GmbH, Gelenau, Germany). For reliable MEP amplitudes, the corticospinal excitability was evaluated by averaging two sets of 20 MEPs with a brief break in between [15]. As body height exerts an influence on MEP measurements, the body height of our subjects was recorded and compared between groups. The average height in the E1-rTMS group was 172.2 ± 7.30 cm; in the E1-rSMS group, it was 171.1 ± 7.70 cm; in the E1-sham-spinal group, it was 172.3 ± 6.40 cm; and in the no-stimulation group, it was 171.2 ± 6.60 cm. In the E2-rTMS group, the body height was 175.5 ± 6.45 cm; in the E2-rSMS group, it was 177.5 ± 7.97 cm; and in the E2-sham-spinal group, it was 176.2 ± 6.14 cm. Body height did not differ significantly between groups. It must be considered that we did not calculate the distal motor latency of the peripheral nerves in our study population, which could have influenced the MEP output.

### 2.7. rTMS, rSMS, Spinal Sham Stimulation

Repetitive TMS was applied at 5 Hz with an intensity of 110% of RMT over the hotspot of the right primary motor cortex (M1), and the spinal cord was stimulated because we expected that spinal cord stimulation would require a higher stimulation intensity to induce neural changes. The 5 Hz rTMS was applied in five runs. Each run consisted of five blocks of 50 stimulations per train. A total of 1250 stimulations were delivered in 10 min.

**Figure 2:**
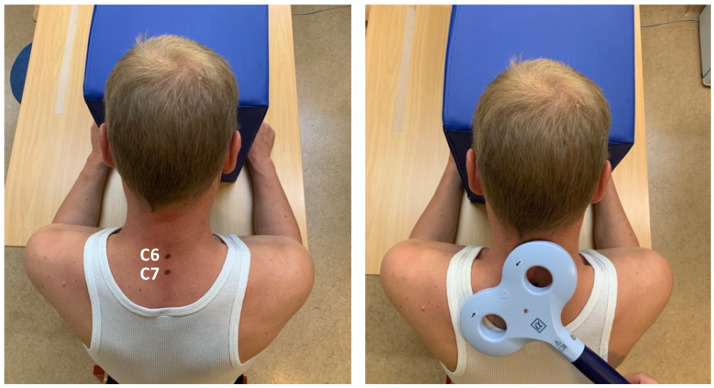
For rSMS, the spinous process of the seventh cervical vertebra (C7) was identified as the most landmark of the skeleton. The center of the coil was positioned below C7 and rotated 45° to the left to stimulate the left CST in its root over the anterior grey matter horn.

According to a recent study, the amplitude of the soleus Hoffmann’s Reflex was influenced by the coil position at TH8 and below but not by the MEP [16]. For the rSMS, we chose to position the coil in E1 and E2, as it is used in routine measurements to determine central motor conduction time. Therefore, the spinous process of the seventh cervical vertebra (C7) was identified as the main landmark of the skeleton. The coil’s center was positioned below C7 and turned 45° to the left to stimulate the left CST in its root over the anterior grey matter horn (Figure 2). The coil’s position was modified until a visible contraction of the hand and fingers resulted from the stimulation target. The coil used for rTMS was also used for rSMS. The stimulation protocol for rSMS was indistinguishable from that for rTMS.

**Figure 3:**
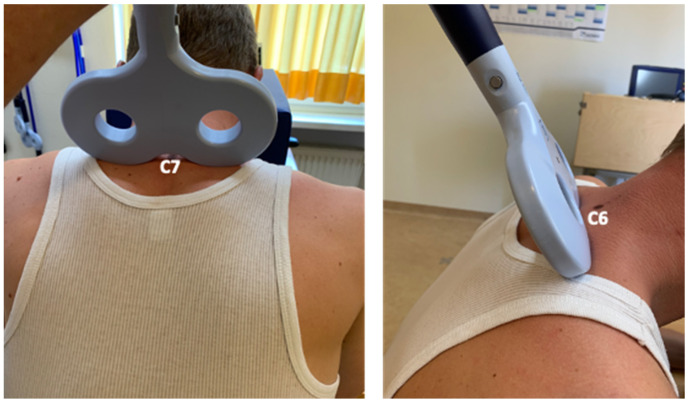
For spinal sham stimulation, the coil is usually rotated 180°. In this current study, a different sham stimulation setting was used because a magnetic field is still induced when the coil is rotated 180°. Therefore, we tilted the magnetic coil by 90° to avoid magnetic stimulation from a 180°-rotated coil.

For spinal sham magnetic stimulation in E1 and E2, the spinous process (vertebra prominens) of the seventh cervical vertebra (C7) was first identified. The coil was positioned under C7 and was tilted 90° anteriorly (Figure 3). The same coil used for rTMS and rSMS was used for spinal sham stimulation in E1 and E2. The stimulation protocol was identical to that used for rTMS and rSMS.

Only in E1 was a group of subjects tested and trained in JTT without any stimulation. In E2, the test and motor training procedure were the same as for the rTMS, rSMS, and sham-spinal group in E1.

### 2.8. Randomization and Blinding

Participants were randomized into groups using sequentially numbered, opaque sealed envelopes (SNOSE) [17].

One person carrying out the motor tests was blinded to the group assignment of the participants. Another person who administered the stimulation was not involved in the motor testing and motor training. The person who conducted the motor training was also blinded to the participants’ affiliation. The data analyst had no knowledge of the group allocation. Participants were blinded as to whether they received real or sham stimulations.

### 2.9. Sample Size

The sample size was determined using the “G*Power software” (Universität Düsseldorf: Psychologie–HHU; version 3.1) [18]. A comparable study has not yet been published. Therefore, the effect size was estimated based on a previous study [19] that demonstrated a consistent increase in MEP amplitude with 5 Hz rTMS. We expected a lower effect size of 1.1, and the calculated sample size was 12 in each group (α error probability of 0.05 with a power (1-β error probability) of 0.80). 

### 2.10. Outcomes, Data Analysis, and Statistical Procedures

All statistical calculations were carried out using IBM SPSS statistics 29 (IBM, Armonk, NY, USA). 

A normal distribution was tested with the Kolmogorov–Smirnov Test. Levene’s test was applied to assess the equality of variances across the groups. 

For E1 and E2, mixed design analyses of variance (ANOVA) were performed to test for differences in the repeated measures of JTT with each hand between groups. In these mixed design ANOVAs, Group was the independent factor, and JTT (pre- and post-test) and Hand (left and right) were the dependent factors. Separate mixed design ANOVAs were applied for the repeated measures RMT, Amplitude and Latency of MEP as dependent factors (pre- and post-test), and Group as an independent factor. Post hoc analyses were performed for significant main effects and interactions.

One-way ANOVAs were used to ensure that the baseline values of the JTT non-dominant left hand (the training hand) at pre-test were not statistically significant between experimental groups. 

For the within-group analysis (pre- and post-test), a paired t-test was used if the normal distribution was given; otherwise, the Wilcoxon test was used. 

For E1 and E2, the level of statistical significance was set at *p* < 0.05, and the Bonferroni correction was used for multiple comparisons. 

Motor skill represents improvement in the motor learning paradigm [20]. We were interested in the gain in motor training performance in combination with repetitive magnetic stimulation. We therefore calculated the effect size based on the percentage change (from pre- to post-test) in the JTT left hand (%JTT = (JTT_post_ − JTT_pre_)/JTT_pre_) × 100%) using Cohen’s d according to the following formula: mean difference between two groups divided by the result of the pooled standard deviation. The effect size is defined as small (0.2), medium (0.5), and large (0.8) [21].

## 3. Results

Out of 96 subjects, 93 subjects completed E1 and E2. One subject from E1-sham-spinal and two subjects from E2-sham-spinal did not complete the experiment.

### 3.1. E1: JTT Pre- and Post-Test Results

- The baseline values of JTT left hand were not significantly different between groups (F (3,55) = 1.95; n.s.).
- There was a significant main effect for JTT (F (1,55) = 274.91; *p* < 0.001) showing a motor learning effect across all groups.

**Figure 4:**
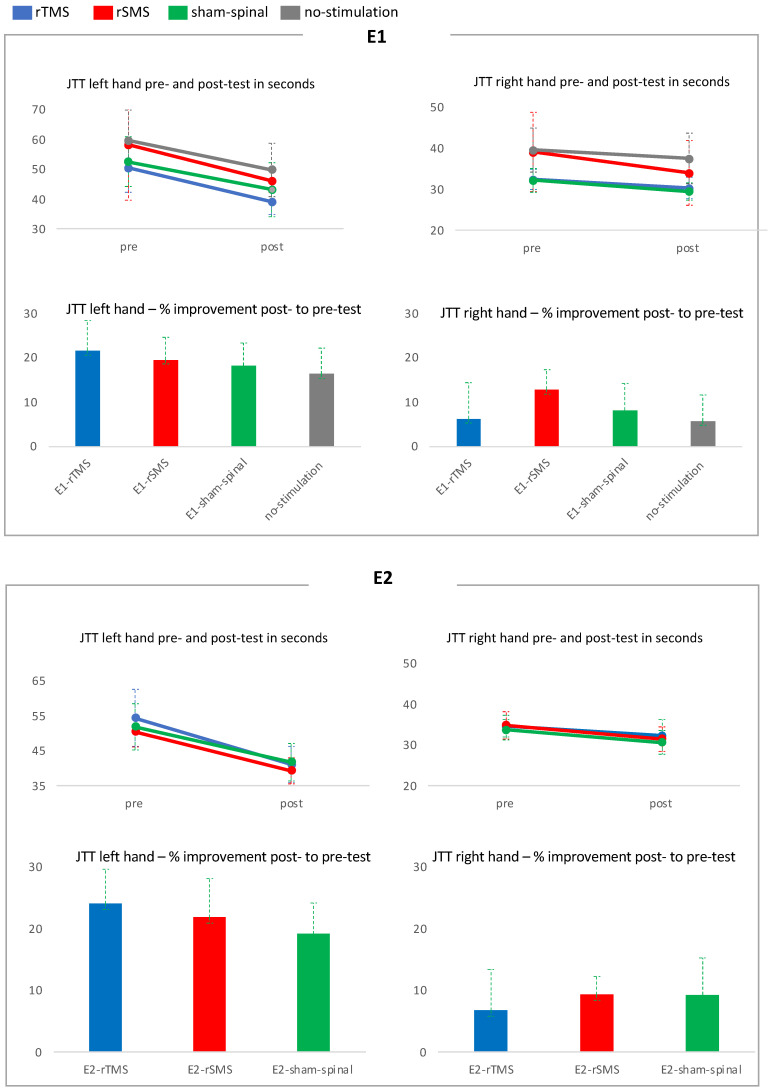
Pre- and post-test in seconds of the JTT, with the left and right hand displayed, as well as percent improvement of JTT from the pre- to post-test for E1 and E2.

As expected, a significant main effect was also found for Hand (F (1,55) = 319.99; *p* < 0.001), indicating higher test performance with the dominant right hand.
The interaction between JTT and Group was not significant (F (3,55) = 0.69; n.s.).
The interaction between Hand and Group was not significant but showed a trend (F (3,55) = 2.31; *p* = 0.086).
The interaction between JTT and Hand was significant (F (1,55) = 201.75; *p* < 0.001).
Thus, the motor performance gain was dependent on laterality.
The three-way interaction between JTT, Hand, and Group was not significant (F (3,55) = 1.36; n.s.), (Table 1, Table 2 and Figure 4).

- Between-group analysis: JTT left and right hand showed no significant difference between groups.
- Within-group analysis: Paired *t*-test showed a significant increase for the left and right hand in all groups (Table 3).

- Effect sizes: The effect size of E1-rTMS was large (d = 0.8) compared to the no-stimulation group, medium compared to E1-sham-spinal (d = 0.6), and small compared to E1-rSMS (d = 0.4). The effect size of E1-rSMS was small compared to E1-sham-spinal (d = 0.3) and medium compared to the no-stimulation group (d = 0.6). The effect size of E1-sham-spinal was small compared to the no-stimulation group (d = 0.4) (Table 4).

### 3.2. E1: RMT, Amplitude and Latency

- There was no significant main effect for RMT (F (1,55) = 0.04; n.s.).

The interaction between RMT and Group was not significant (F (3,55) = 0.69; n.s.).
There was no significant main effect for Latency (F (1,55) = 1.97; n.s.).
The interaction between Latency and Group was not significant (F (3,55) = 0.12; n.s.).
The main effect for Amplitude (F (1,55) = 7.03; *p* = 0.01), as well as the interaction between Amplitude and Group, were significant (F (3,55) = 2.98; *p* = 0.04).

- Between-group analysis: MEP amplitude was not significantly different between groups.
- Within-group analysis: A significant enhancement in MEP amplitude within the rSMS group (*p* < 0.02) was found.

### 3.3. E2: JTT Pre- and Post-Test Results

- The baseline values of JTT left hand were not significantly different between groups (F (2,31) = 1.10; n.s.)
- There was a significant main effect for JTT (F (1,31) = 339.41; *p* < 0.001). Thus, all subjects improved their motor performance between pre- and post-test periods.

A significant main effect was also found for Hand (F (1,31) = 343.24; *p* < 0.001), indicating that subjects performed better with their dominant right hand.
The interaction between JTT and Group was not significant (F (2,31) = 0.81; n.s.).
The interaction between Hand and Group was not significant (F (2,31) = 1.52; n.s.).
The interaction between JTT and Hand was significant (F (1,32) = 158.16; *p* < 0.001). Thus, the motor performance gain was dependent on laterality.
The three-way interaction between JTT, Hand, and Group was significant (F (2,31) = 3.40; *p* < 0.046). Thus, in E2, the influence of group affiliation, hand laterality, and motor learning were somewhat interdependent (Table 5, Table 6 and Figure 4).

- Between-group analysis: JTT left and right hand showed no significant difference between groups.
- Within-group analysis: Paired *t*-test showed a significant increase for the left and right hand in all groups (Table 7).

- Effect sizes:

The effect size of E2-rTMS was large (d = 0.9) compared to the E2-sham-spinal group and was small compared to E2-rSMS (d = 0.4). The effect size of E2-rSMS was medium compared to E2-sham-spinal (d = 0.5) (Table 8).

### 3.4. E2: RMT, Amplitude and Latency

- There was no significant main effect for RMT (F (1,31) = 0.62; n.s.).

The interaction between RMT and Group was not significant (F (2,31) = 0.26; n.s.).
There was a significant main effect for Latency (F (1,31) = 4.19; *p* = 0.049). Latency was shorter for the post-measurement of the MEP.
The interaction between Latency and Group was not significant (F (2,31) = 0.98; n.s.).
There was no significant main effect for Amplitude (F (1,31) = 1.89; n.s.).
The interaction between Amplitude and Group was not significant (F (2,31) = 0.34; n.s.).

- Within group analysis: The latency was only substantially reduced in the rSMS group from the pre- to post-test (*p* < 0.04).

## 4. Discussion

A direct comparison between repetitive cortical and trans-spinal stimulation combined with the same motor training was needed to assess the magnitude of their effect sizes. Therefore, in two experiments (E1 and E2), we analyzed the effect of non-invasive stimulation on the same motor training applied to different parts of the body.

In E1, rTMS (cortical impact), rSMS (spinal interference), and spinal sham stimulation (sham-spinal, peripheral inflow) were applied once in combination with motor training, while one group received the same motor training only (without stimulation). In E2, rTMS, rSMS, and sham-spinal were applied in combination with motor training over several days. 

We found no significant difference in JTT left hand between the groups in either E1 or E2. As no comparable study had been published previously, the exact effect size was unknown, and a precise power calculation could not be performed. Retrospectively, considering the effect size between groups, our sample size was too small (underpowered) to detect a significant difference between the groups. However, if the effect size is taken into account, we found that rTMS had the greatest effect on motor training, followed by rSMS and then sham-spinal. Daily stimulation led to additional training gains. To our knowledge, this is the first study to compare the effects of rTMS and rSMS with the same motor training content. The results of this study suggest that rSMS increases excitability and also enhances motor training performance. It warrants further studies to discover whether patients who cannot be effectively treated with rTMS would benefit from rSMS. 

### 4.1. The Effect of rTMS

In this current study, high-frequency rTMS was applied to the primary motor cortex contralateral to the trained left hand. Training gain was superior with rTMS compared to rSMS, sham-spinal, and motor training alone (no-stimulation group in E1). Cohen’s effect size of training gain was large when rTMS was added to the same motor skill training when it was executed without any stimulation (no-stimulation group in E1). Interestingly, this difference appeared even after a single stimulation. With daily stimulation (in E2), there was even a large effect size between E2-rTMS and E2-sham-spinal, confirming the effect of rTMS to enhance motor training. In JTT, the subjects practiced movements that partly simulated everyday life and were more complex in their execution; so that a network of several regions was therefore involved in the motor training process of JTT. Following a previous report [22], we therefore suggest that rTMS activates a network rather than the area under the stimulation coil. This is supported by studies showing that rTMS over a specific region influences the hemodynamic response in remote motor regions [23,24,25,26]. However, it is important to consider that increasing excitability with rTMS within an inhibitory balanced network disrupts the underlying network and worsens motor training [27]. In this current study, high-frequency rTMS over the primary motor cortex induced a beneficial enhancement of motor training after a single stimulation (E1) and after repeated stimulation over the following days (E2), although we did not find an excitatory increase in the measurement of MEP. In recent years, studies have demonstrated a high individual variability of MEP amplitude after rTMS and have shown that there is no general increase after excitatory stimulation with high-frequency rTMS and anodal transcranial direct current stimulation [28,29,30,31,32,33]. This interindividual variability has been suggested to depend on several factors such as stimulation time of day [34], TMS coil orientation [35], genetic variation [36], sample size for a between-group analysis [29,32,37], hormonal changes during the menstrual cycle [38], anatomical aspects such as cortical thickness [39], instantaneous brain state at the time of stimulation [34,40,41,42], and other factors [43]. In this current study, the stimulation protocol was standardized to account for several variables that influence inter-individual variability (same stimulation time per day, same orientation of the TMS coil, and time of the menstrual cycle). However, other aspects of individual variability could account for the high variability of MEP results in the rTMS group. Other limitations of our studies are that there are different methods of TMS and that we did not use a navigation system for the application of TMS. Navigated TMS is recommended to achieve better stimulation results. In addition, electric-field-navigated TMS produces a significantly higher rate of positive responses than line-navigated TMS [44]. The results of our study should be interpreted with caution. The generalizability of our findings may be limited because the effect sizes were calculated based on the specific motor training program and stimulation method used in our study. Different stimulation approaches, motor programs, and training durations may have resulted in different effect sizes. 

### 4.2. The Effect of Spinal Sham Stimulation

Considering the effect size in E1, we found a higher training gain with E1-sham-spinal as compared to the no-stimulation group. This training improvement may be surprising, but we believe that the way we performed the sham stimulation had an additional afferent effect. Normally, in sham stimulation, the coil is rotated 180° to produce the same magnetic stimulation tone and to maintain the sensation of the coil on the body. We decided to use a different sham stimulation setting because a magnetic field is still induced when the coil is rotated 180°. We therefore tilted the magnetic coil by 90° to avoid magnetic stimulation from a 180° rotated coil. However, even with the modified coil orientation, there was an additional effect. Either the local pressure of the coil, and/or the magnetic influence from the front of the coil, may have caused an afferent stimulation. This supports a previous study showing that there is no placebo effect of TMS on motor learning [3].

### 4.3. The Effect of rSMS and Prospects

A notable finding was that rSMS had an enhancing effect on motor training performance in both E1 and E2 as compared to the E1- and E2-sham-spinal and no-stimulation group of E1. Cohen’s effect size was superior to the sham-spinal and no-stimulation groups. The use of rSMS to increase excitability within the CST [8] with an additional effect on motor training is noteworthy. This may be relevant to some clinical conditions such as stroke. The degree of recovery in stroke patients depends in part on the integrity of the CST, which determines the recruitment of brain regions associated with recovery [45]. Recovery from stroke is better when a motor-evoked potential (MEP) can be elicited by TMS [46,47]. The potential for recovery of upper limb function in patients without an evoked MEP is severely limited when the fractional anisotropy (FA) ratio (between ipsilesional and contralesional) of the CST, as acquired by diffusion tensor imaging, is less than 0.15 [48]. Consequently, significant damage to the CST limits recovery after stroke. We can therefore assume that excitability-enhancing high-frequency rTMS over ipsilesional M1 will not significantly improve performance in these patients because the output signal to spinal cord neurons is significantly reduced. This is consistent with reports that not all stroke patients benefit from high-frequency rTMS over ipsilesional M1 [49]. The question then arises as to whether additional performance benefits can be achieved with rSMS in these patients.

With rSMS, paravertebral muscles and paravertebral nerves are stimulated, and these afferent impulses have an influence on cortical activation [6,7]; consequently, patients with relapsing–remitting multiple sclerosis [5,50,51] and Parkinson’s disease [52] have benefited from rSMS in terms of balance, functional walking, reduced spasticity, and reduced risk of falls. The amplitude of the Hoffmann’s Reflex of the soleus muscle was observed to decrease following rSMS of eight thoracic vertebrae, while the MEP amplitude remained unchanged. This suggests that rSMS also influences the presynaptic level [17]. In this study, rSMS was found to facilitate CST. It is possible that different stimulation approaches, such as variations in stimulation intensity and duration, coil position, stimulation level, and MEP derivation, may account for the different results in MEP amplitude. 

Further investigation is required to determine whether the increase in CST excitability was solely due to afferent stimulation (as an afferent–efferent loop model), as demonstrated by epidural spinal cord stimulation of the cervical dorsal roots [53], or whether efferent pathways were also stimulated. If rSMS is able to stimulate the CST (e.g., the grey matter neurons in the anterior horn [54]) in addition to afferent stimulation, then sub-lesional rSMS has the potential to aid recovery in patients with extensive CST damage, e.g., after stroke. This current study may provide a basis for further research.

## 5. Conclusions

This study suggests that rSMS has the potential to improve the performance of MT. Further research is now needed to determine whether certain groups of patients (who cannot be treated effectively with rTMS) would benefit from the addition of rSMS.

## Figures and Tables

**Table 1:** Mean time pre- and post-test in seconds (s) for the JTT with the left hand with standard deviations (E1). The time decrease from post-test to pre-test was calculated as an increase in %JTT left hand (with standard deviations). The mean %JTT of the left hand of the E1-rTMS group increased the most with 21.6 ± 6.7%, then E1-rSMS with 19.5 ± 5.1%, followed by the E1-sham-spinal group with 18.2 ± 5.1%, and finally the no-stimulation group with 16.3 ± 5.8%.

E1 Groups	Mean Time Pre-Test	Mean Time Post-Test	Increase in %
E1-rTMS	50.4 ± 8.1 s	39.1 ± 4.4 s	21.6 ± 6.7%
E1-rSMS	58.1 ± 18.5 s	46.1 ± 3.8 s	19.5 ± 5.1%
E1-sham-spinal	52.5 ± 8.4 s	43.2 ± 9.0 s	18.2 ± 5.1%
no-stimulation	59.6 ± 10.2 s	49.8 ± 8.9 s	16.3 ± 5.8%

**Table 2:** Mean time pre- and post-test in seconds (s) for the JTT with the right hand with standard deviations (E1). The time decrease from post-test to pre-test was calculated as an increase in %JTT right hand (with standard deviations). The mean %JTT for the right hand was 6.2 ± 8.2% for E1-rTMS, 12.8 ± 4.4% for E1-rSMS, 8.1 ± 6.0% for E1-sham-spinal, and 5.7 ± 5.8% for the no-stimulation group.

E1 Groups	Mean Time Pre-Test	Mean Time Post-Test	Increase in %
E1-rTMS	32.4 ± 2.5 s	30.3 ± 2.7 s	6.2 ± 8.2%
E1-rSMS	39.1 ± 9.7 s	34.0 ± 7.9 s	12.8 ± 4.4%
E1-sham-spinal	32.2 ± 2.9 s	29.5 ± 2.1 s	8.1 ± 6.0%
no-stimulation	39.6 ± 5.4 s	37.5 ± 6.2 s	5.7 ± 5.8%

**Table 3:** The significance level (*p*) of the paired *t*-test (pre- and post-test) of E1 groups for JTT. Left and right hand.

E1 Groups	JTT Left Hand	JTT Right Hand
E1-rTMS	4.0 × 10^−7^	0.009
E1-rSMS	1.2 × 10^−6^	1.6 × 10^−6^
E1-sham-spinal	2.0 × 10^−8^	0.0003
no-stimulation	1.8 × 10^−7^	0.002

**Table 4:** Cohen’s d. Effect size of %JTT left hand between E1 groups.

E1 Groups	E1-rTMS	E1-rSMS	E1-Sham-Spinal
E1-rTMS	-	-	-
E1-rSMS	0.4	-	-
E1-sham-spinal	0.6	0.3	-
no-stimulation	0.8	0.6	0.4

**Table 5:** Mean time pre- and post-test in seconds (s) for the JTT with the left hand with standard deviations (E2). The time decrease from post-test to pre-test was calculated as an increase in %JTT left hand (with standard deviations). The mean of %JTT left hand increased the most in the E2-rTMS group with 24.1 ± 5.6%, followed by E2-rSMS with 21.9 ± 6.3%, and finally the E2-sham-spinal group with 19.2 ± 5.0%.

E2 Groups	Mean Time Pre-Test	Mean Time Post-Test	Increase in %
E2-rTMS	54.4 ± 8.2 s	41.1 ± 5.1 s	24.1 ± 5.6%
E2-rSMS	50.5 ± 4.4 s	39.4 ± 3.7 s	21.9 ± 6.3%
E2-sham-spinal	51.9 ± 6.6 s	41.8 ± 5.3 s	19.2 ± 5.0%

**Table 6:** Mean time pre- and post-test in seconds (s) for the JTT with the right hand with standard deviations (E2). The time decrease from post-test to pre-test was calculated as an increase in %JTT right hand (with standard deviations). The mean %JTT for the right hand was 6.8 ± 6.6% for E2-rTMS, 9.4 ± 2.9% for E2-rSMS, and 9.3 ± 6.0% for E2-sham-spinal.

E2 Groups	Mean Time Pre-Test	Mean Time Post-Test	Increase in %
E2-rTMS	34.6 ± 2.7 s	32.3 ± 3.9 s	6.8 ± 6.6%
E2-rSMS	34.8 ± 3.4 s	31.5 ± 3.0 s	9.4 ± 2.9%
E2-sham-spinal	33.7 ± 2.5 s	30.6 ± 2.9 s	9.3 ± 6.0%

**Table 7:** The significance level (*p*) of the paired *t*-test (pre- and post-test) of E2 groups for JTT. Left and right hand.

**E2 Groups**	**JTT Left Hand**	**JTT Right Hand**
E2-rTMS	3.1 × 10^−8^	0.0007
E2-rSMS	1.0 × 10^−9^	2.6 × 10^−7^
E2-sham-spinal	3.1 × 10^−6^	1.3 × 10^−5^

**Table 8:** Cohen’s d. The effect size of %JTT left hand between E2 groups.

E2 Groups	E1-rTMS	E1-rSMS
E2-rTMS	-	-
E2-rSMS	0.4	-
E2-sham-spinal	0.9	0.5

## Data Availability

Data are available on request from the corresponding author.
